# Stricture of the Urethra of Twenty Years’ Standing; External Incision without Guide

**Published:** 1855-01

**Authors:** Henry Lee


*Stricture of the Urethra of Twenty Years' Standing; External Incision without Guide.* By Mr. HENRY LEE.—This patient is a hair weaver, about forty years of age, of a thin, sickly look, and observed the first symptoms of stricture twenty years before his present admission. At that period retention of urine suddenly came on, when a personal friend of the patient, seeing the state he was in, shaped a piece of *whalebone from an umbrella* into the form of a bougie, and passed it up the urethra. Immediately after the operation, the urine flowed copiously.

The patient now remained well for three or four years, when symptoms of stricture again presented themselves, but not to such a degree as to cause him much inconvenience, the use of the catheter being, however, now and then required.

Four years before his admission here, the distress from the stricture became suddenly so great as to induce Mr. Guthrie, under whose care he then was, to divide the stricture with Stafford's instrument. After this operation, the patient passed his urine freely, for a short time, but soon the stricture returned, and he has been suffering from it more or less up to his admission. When first seen in the hospital, September 14th, 1854, there was pain in the lower part of the abdomen, and across the loins, and the bladder could be distinctly felt, through the parietes of the abdomen, to be hard and full. There was total inability to pass urine, which fluid only came in drops. The endeavors to introduce a catheter, both without and within the hospital, having proved useless, Mr. Lee had the patient brought into the theatre, passed down an instrument as far as the obstacle, and with no better guide than the end of the catheter, freely divided the urethra. When this perineal section was accomplished, No. 6 catheter was introduced into the bladder, and there retained for five days.

The urine a few days afterwards passed both through the wound and the urethra, and the patient after remaining several weeks in the hospital is on the point of being discharged. The wound is all but healed, and scarcely any urine escapes through the perinæum.

Mr. Lee, in a clinical lecture on this case, after giving a sketch of its principal features; and having pointed out the different kinds of unpleasant consequences following stricture, and the various methods which have been devised for the cure of coarctation, said :—

"The plan of dividing stricture by instruments passed down the urethra have been variously modified; sometimes an instrument of the shape of a trocar has been made to protrude from the end of a catheter, so as to perforate the stricture; sometimes the instrument used has been made in the shape of a lancet, and sometimes a long very thin knife has been passed down to the stricture upon a director. Another plan has lately been adopted in France, where an instrument could be introduced into the bladder. The instrument used resembles in construction a lithotrite, but the blades are capable of being separated from each other to the extent of about a quarter of an inch. The instrument is then introduced, and the blades suddenly separated, so as forcibly to tear open the stricture.

Now all these plans have the disadvantage, besides that of endangering the vitality of a portion of the mucous membrane of the canal, of not providing an escape for any portion of urine which may flow from the urethra through the opening which they make. Under these circumstances it has been proposed to make an incision in the perinæum, and to divide the stricture from without. This plan, of which I now propose to speak, has at least the advantage, when properly performed, of being free from the dangers arising from making false passages, extravasation of urine, and purulent deposits.

The cases for which this operation has been recommended are of three kinds :—

1. Where the stricture presents an extreme degree of irritability, and resents, by violent local and constitutional disturbance, any efforts to produce dilatation.

2. Cases in which the stricture, when dilated, rapidly contracts again.

3. Cases in which, after the dilatation of the passage, micturition is nevertheless painful, difficult, and uncertain.

To these three classes we may add a fourth, not admitted by some surgeons—namely, cases in which no instrument can be passed into the bladder.

For these affections a free incision of the contracted part of the urethra has been maintained to be the proper mode of treatment, and to be in fact required.

The mode of performing this operation, as described by Mr. Syme, is as follows:

A grooved director is first introduced through the stricture, where this can be done, (and Mr. Syme is of opinion that, with care and attention, there is no stricture through which an instrument may not be made to pass.) The patient then being placed upon his back, at the edge of the table, with his legs bent, as in the operation for lithotomy, an incision, about one inch and a-half in length, is made exactly in the raphe of the perinæum. The whole of the thickened, indurated, and contracted texture is then divided upon the director, to the extent of an inch or two, or more if necessary. A No. 8 silver catheter is then passed into the bladder, and allowed to remain there for at least two, and not more than three days.

Great stress is laid upon the fact of the incision being made exactly in the middle line of the perinæum, in order to avoid the artery of the bulb which lies by the side of the canal.

The only sources of danger alleged to exist are hæmorrhage and extravasation of urine. But if the knife is kept exactly in the median line, the only vessels that are likely to bleed are the smallest branches of the superficial perineal artery, and the cells of the corpus spongiosum; the bleeding from these may be checked, should it be desirable, by placing a piece of folded lint between the edges of the wound, and applying the slightest degree of pressure for a few hours.

The liability to extravasation of urine after this operation, I will presently consider, reserving it for separate remarks, as I conceive the liability or otherwise to its occurrence forms the grand distinction as to this operation being admissible or not.

After the operation of perineal section, the catheter is tied in the bladder, and the patient put to bed. At the end of forty-eight hours the catheter may be removed. A full-sized bougie should be introduced once a week for three or four weeks, and then at more distant intervals, according to circumstances.

Now this operation, as I have described it, appears a very simple affair, and if this were all, there is little doubt which is to be preferred, the pain and inconvenience, (to say nothing of the danger of a stricture) or the simple operation of dividing a portion of the urethra on a grooved director. But, unfortunately, the operation has not been found to be one of such a very simple nature in its consequences, even where an instrument could be got into the bladder, much less in cases where no staff could be passed.

Mr. Syme, of Edinburgh, asserts that he has performed this operation a great number of times without any ill effects, excepting only some consequent symptoms of nervous irritation. But in other hands the most serious mischief has often supervened. Patients have been attacked with a shivering fit the day after the operation; there has been a quick, irritable condition of the pulse, accompanied, perhaps, by profuse perspiration, and want of sleep. These symptoms have continued, the patient's tongue becoming brown and coated, and, in a certain number of cases, the patient has died. On a post mortem examination, some purulent infiltration has generally been found about the neck of the bladder, or some secondary inflammation in other parts.

Now how are we to account for such different results in the practice of various surgeons of equal skill? I believe that something like a solution may be arrived at by an attentive consideration of the description which Mr. Syme has given of the operation.

In the way in which he performs it, the only fascia concerned, as he says, is that which lies immediately under the integuments. In other words, he divides only the skin, superficial fascia, and the urethra. Now it is clear from this description, that all the strictures which he has operated upon have been situated anterior to the membranous portion of the urethra; for had he operated upon any stricture in the membranous portion of the canal, even though situated quite at its anterior part, he must have been in danger of wounding the deep perineal fascia as well as the superficial. Hence, then, arises a practical distinction of the utmost importance. When a stricture is situated in the bulb of the urethra, it may be divided from without, and any urine which escapes from the passage is sure to pass out at the external wound. But the circumstances are different when the knife, in passing along the grooved director or sound, perforates the deep perineal fascia, and wounds the urethra as it passes through this part. The urine which escapes from the passage may then lodge in the wound made in the deep perineal fascia, and a drop or two may become infiltrated behind this dense structure. It will there give rise to inflammation, and having no means of escape, will produce violent constitutional irritation. When once inflammation is established in the cellular tissue of this part, its products will permeate the areolar tissue, and may thus propagate the inflammation to the outside of the bladder, and the cellular tissue within the pelvis, thus giving rise to the abscesses and the purulent infiltration which I have mentioned.

But where the incision is confined to the superficial fascia, and to the bulb of the urethra, there is, as I have said, very little danger of any of these accidents occurring; and by choosing his cases, (as it were,) and confining himself to those in which the stricture is situated anterior to the membranous part of the urethra, Mr. Syme has met with the success to which I have alluded.

Hence there appear to be two classes of cases; those in which the stricture is situated in that part of the urethra corresponding to the corpus spongiosum, and those in which the stricture is at the anterior part of the membranous portion of the urethra. In the former situation, as far as our present evidence goes, the stricture may be divided with comparative impunity; in the latter, most severe and even fatal symptoms have followed.

But practically, it will be asked, how are we to know that a stricture is confined to the bulb of the urethra, or to any part in front of this? Where an instrument can be introduced, the point may be made out in this way, and Mr. Syme never operates unless he is able first to introduce an instrument into the bladder.

In the case to which I have drawn your attention, I was led to believe that I might with impunity divide the stricture, from the circumstance of Mr. Guthrie having divided it by internal incision before, without any ill effects. This proved to me, either that the stricture was confined to the bulb of the urethra, or, if situated farther back, that the surrounding parts were so consolidated by inflammation as to preclude the danger which might arise from infiltration of urine."—*Lon. Lan.*

